# Reshaping Emergency Care: Dynamics of OHCA and STEMI in a Three-Year Analysis

**DOI:** 10.3390/epidemiologia5030026

**Published:** 2024-07-09

**Authors:** Francesca Bottega, Erika Kacerik, Gabriele Perotti, Carlo Signorelli, Giuseppe Ristagno

**Affiliations:** 1 Faculty of Medicine, School of Public Health, University of Vita-Salute San Raffaele, Via Olgettina 60, 20132 Milano, Italy; 2 Agenzia Regionale Emergenza Urgenza Headquarters (AREU HQ), Via Campanini 6, 20124 Milano, Italy; 3 Dipartimento di Fisiopatologia Medico-Chirurgica e dei Trapianti, Via Festa del Perdono 7, 20122 Milano, Italy; 4 Fondazione IRCCS Ca’ Granda Ospedale Maggiore Policlinico, 20090 Milano, Italy

**Keywords:** emergency department, COVID-19, OHCA, STEMI, Lombardy region

## Abstract

The COVID-19 pandemic drastically reshaped healthcare in Lombardy, Italy, notably impacting EMS and emergency departments and altering the epidemiology of time-dependent pathologies such as STEMI and OHCA. While previous studies focused on modifications during the pandemic peak, with an increase in the number of OHCA diagnoses and a reduction in the number of STEMI, little evidence exists regarding the inter-pandemic phases. We analyzed OHCA and STEMI accesses to the ED in the regional emergency department access register (EUOL) for 2019–2021. The analysis revealed a significant difference in monthly diagnosis averages. For STEMI, the change was statistically significant (F(2, 33) = 4.45, *p* = 0.02), while it was not for OHCA (F(2, 33) = 0.18, *p* = 0.83). Despite the monthly decreases, the likelihood of receiving a STEMI diagnosis increased with total accesses, OR 1.40 [95% CI 1.25–1.58, *p* < 0.0001]. Additionally, there was a significant increase in March 2020 discharge diagnoses for OHCA compared to March 2019, OR 3.35 [95% CI 2.88–3.90, *p* < 0.0001], corresponding to the first pandemic wave. Therefore, our analysis indicates that the epidemiology of STEMI and OHCA was altered during the COVID-19 pandemic.

## 1. Introduction

Italy was the first European country involved in the COVID-19 pandemic [1,2], and Lombardy was the Italian region most severely affected by the pandemic. During the first wave, the Lombardy death toll accounted for approximately half of the total deaths recorded across the entire Italian territory [3]. COVID-19 had a great impact on the healthcare system and emergency medical services (EMS) system [4,5], as both had to adjust their organization due to the pandemic [6]. COVID-19 has indeed modified all aspects of emergency–urgency care. There have been changes in professional training [7], in the number of accesses to the emergency department (ED) and transport modalities of the EMS system [3,8], and in the clinical characteristics of patients [3]. With regards to the EMS system, there has been a change in the diagnosis of time-dependent pathologies [9], probably caused by the stay-at-home policy, for which patients postponed access to the ED [10], and on human activities during the restrictions, with a consequent change in risk factors [11,12]. 

Out-of-hospital cardiac arrest (OHCA) is the cessation of cardiac activity in a non-hospital setting. It is a serious phenomenon, with an incidence rate of 1 case per 1000 inhabitants and a survival rate of less than 10% [13,14], contributing to 60–70% of all cardiovascular deaths in Italy [14,15]. The return of spontaneous circulation (ROSC), indicative of the restoration of spontaneous cardiac activity, is influenced by various factors, including the timing of medical assistance, the level of first aid training among bystanders, and the emergency system protocol employed [16]. However, effective resuscitation maneuvers are crucial for achieving ROSC. The COVID-19 pandemic has led to an increase in the incidence and mortality of OHCA, with a 60% rise compared to 2019 [17]. One contributing factor is the decreased involvement of bystanders in initiating cardiopulmonary resuscitation maneuvers during the pandemic, which was driven by psychological concerns regarding the risk of viral exposure [16,18].

From the literature data, it emerges that Acute ST-Elevation Myocardial Infarction (STEMI) has also been affected by the COVID-19 pandemic. Indeed, there has been a reduction in the number of STEMI cases diagnosed in the ED [19,20,21], a decrease of 40% in admissions with a STEMI diagnosis compared to 2019 [22], and a decline in the number of procedures performed [23]. Furthermore, starting from May 2021, the reduction in the number of diagnoses, when compared to 2019, has no longer been recorded [24]. On the other hand, an increase in the response times of emergency vehicles has been observed [23,25,26].

The Lombardy region is the most inhabited Italian region, with about 10 million residents; there are 218 hospitals, 102 of which are equipped with an ED, and the territorial emergency–urgency (EU) system is coordinated by AREU (“Agenzia Regionale Emergenza Urgenza”—Regional Emergency Urgency Agency), a single institution that coordinates the whole emergency transport system [27]. The Italian EMS is also known as 118, which, historically, was the number to dial for requiring emergency health assistance before the universal emergency phone number of 112 was introduced. Thanks to its organization, AREU manages over 1 million calls and 800,000 dispatches of vehicles per year [13]. During the different pandemic phases, the AREU system had to react quickly through the implementation of protocols for the management of COVID-19 patients and the increase in the number of rescue vehicles, in order to guarantee pre-pandemic first response standards [28,29,30,31]. All of the EDs in the Lombardy region register the accesses on one single portal, called EUOL (“Emergenza Urgenza OnLine”—Emergency Urgency Online) [32]. Within the portal, the accesses to the 102 EDs of the Lombardy region are recorded, and the data are anonymized.

Our study analyzes the emergency department admissions in the largest and most populous Italian region, considering a period of three years. This allows for the examination of three pandemic waves and the inter-pandemic phases.

## 2. Materials and Methods

This is a retrospective observational cohort study. All information was anonymous and there were no sensitive patient data.

We analyzed data included in the EUOL register for the years 2019–2021, taking into account the number of diagnoses of STEMI and OHCA, the triage on ED inpatients, their demographic characteristics, and their mode of arrival (ambulance, walk-in or helicopter). The diagnosis was established by the medical doctor of the ED department. We selected “410.X” as the diagnosis code for STEMI and “427.5” for OHCA, according to the ICD-9-CM classification [33]. We identified 24.645 instances of STEMI and 10.553 cases of OHCA over the course of the three years under study. As the first, second, and third pandemic waves in Italy, we considered, respectively, March 2020, November and December 2020, and February and March 2021, according to the definition given by the Italian Ministry of Health [1].

The categorical variables are presented as numbers, and the continuous variables are presented as mean and standard deviation. The normality of the continuous variables was assessed using the Kolmogorov–Smirnov test, and they were analyzed by ANOVA test. Relative odds ratios (OR) and confidence intervals (with CI 95%) were calculated. Differences were considered significant when *p* < 0.05; otherwise, they were considered non-significant (NS). The Prism 8.0.1 statistical software (GraphPad Software LLC, San Diego, CA, USA) was used for this analysis.

## 3. Results

Following the purpose of the study, we systematically retrieved all recorded admissions to the EDs within the EUOL database due to STEMI or OHCA cases. It is important to note that the dataset solely includes patients who were presented to the ED and does not encompass all patients managed by the emergency medical service. Additionally, it is imperative to highlight that the total number of ED admissions in the Lombardy region accounts for approximately 3 million annually.

### 3.1. STEMI

Regarding the demographic profile of the patient cohort, both the mean age and the percentage of female patients did not change significantly over the three years. Specifically, the mean age in 2019 was similar to 2020, 68.9 years (SD = 14.1) versus 68.5 years (SD = 13.4), *p* = 0.07, and to that of 2021, 68.9 (SD = 14.1) versus 69.00 years (SD = 13.5), *p* = 0.8. Similarly, there was also no reduction in the proportion of female patients from 2019 (32%) to both 2020 (30%) and 2021 (31%).

To assess patients’ complexity, we analyzed the triage code of classification upon the patient’s arrival at the ED; the codes remained stable over the three years. In 2019, we observed 35% and 48% of patients categorized, respectively, with red (emergency) and yellow (urgency) codes. In the years 2020 and 2021, we recorded a slight increase in red codes, accounting for 36% and 37%, respectively, accompanied by a decrease in yellow codes, totaling 47% and 46%, respectively. 

**Figure 1:**
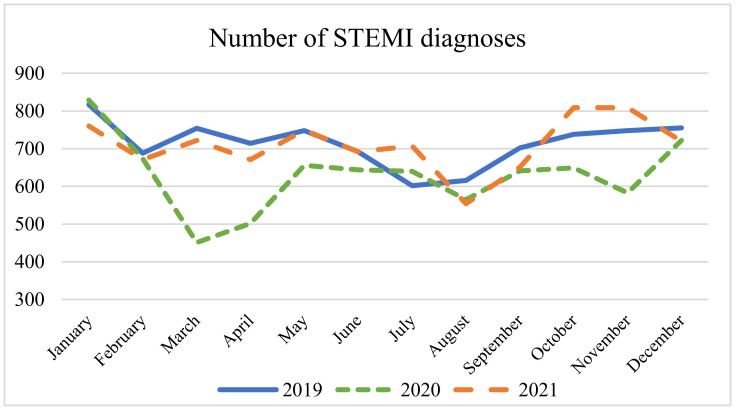
Number of STEMI diagnoses per month in the EDs. ANOVA test: monthly average for 2019 (715) vs. 2020 (630) vs. 2021 (710); *p* = 0.02.

Figure 1 illustrates a substantial decrease in the number of STEMI diagnoses observed during the first and second waves of the pandemic; however, such reduction was not evident during the third wave. The decline in the number of diagnoses during 2020 is significant when compared to 2019; however, the monthly average for 2021 rebounds to levels similar to those observed during 2019. Despite a monthly decline in the absolute number of STEMI diagnoses, there was an increase in the likelihood of receiving a STEMI diagnosis when considering the total number of accesses, with an odds ratio of 1.40, [95% CI 1.25 to 1.58, *p* < 0.0001].

The percentage of patients admitted to the ED and transported via 118 vehicles increased. Specifically, there was a substantial increase noted for the years 2020 (53%) and 2021 (50%) compared to 2019 (46%) (F(2.33) = 12.7, *p* < 0.0001).

**Figure 2:**
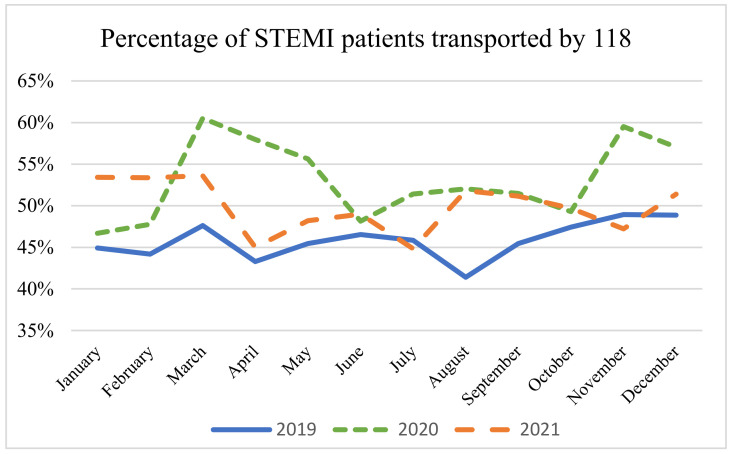
Percentage of patients with STEMI transported by 118. ANOVA test: monthly average 2019 (46%) vs. 2020 (53%) vs. 2021 (50%); *p* < 0.001.

Figure 2 shows that the percentage of STEMI patients transported by the EMS to the EDs of the Lombardy region, has significantly increased. The highest percentage of transported STEMI patients was observed during March 2020, reaching a peak of 61%. Subsequently, there was a marginal decrease during the following waves, with 59% during the second and 55% during the third. By contrast, the monthly percentage of transports carried out by EMS in 2019 stood at 45%.

### 3.2. OHCA

Concerning the demographic characteristics of OHCA patients, we highlighted that both the mean age and the percentage of female patients remained stable over the three years. The mean age in 2019 was comparable to that in 2020, 75.9 (SD = 16.7) versus 75.9 (SD = 16.3), *p* = 0.9 years, and to that in 2021, 76.3 (SD = 13.6) *p* = 0.8 years. Concerning the gender of the patients, there were no notable changes in the proportion of female patients from 2019 (42%) to both 2020 (42%) and 2021 (43%).

**Figure 3:**
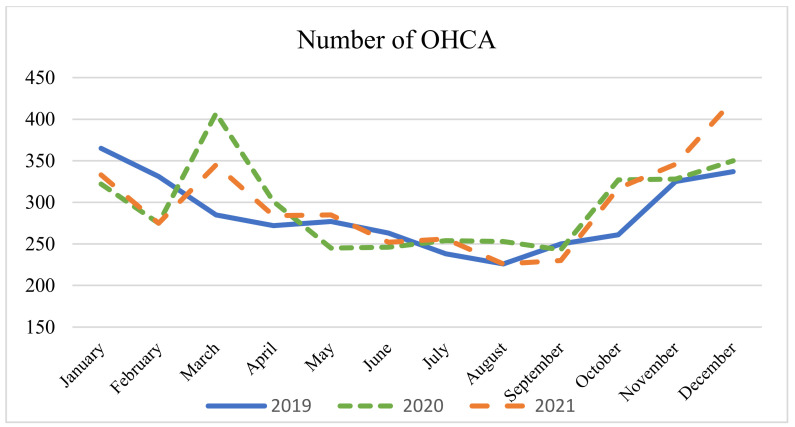
Number of OHCA diagnoses per month in the ED. ANOVA test: monthly average 2019 (286) vs. 2020 (296) vs. 2021 (298); *p* = 0.83.

Figure 3 illustrates the number of OHCA diagnoses registered in the ED. Over the three designated years, the mean number of diagnoses within the ED does not show a statistically significant variation, even if there was an important increase in the number of OHCA diagnoses during the first pandemic wave. However, when comparing March 2019 and March 2020 to the total number of accesses to the ED, there is a considerable elevation in the probability of having a patient discharged with a diagnosis of OHCA in 2020 [OR 3.35, 95% CI 2.88–3.90, *p* < 0.0001].

**Figure 4:**
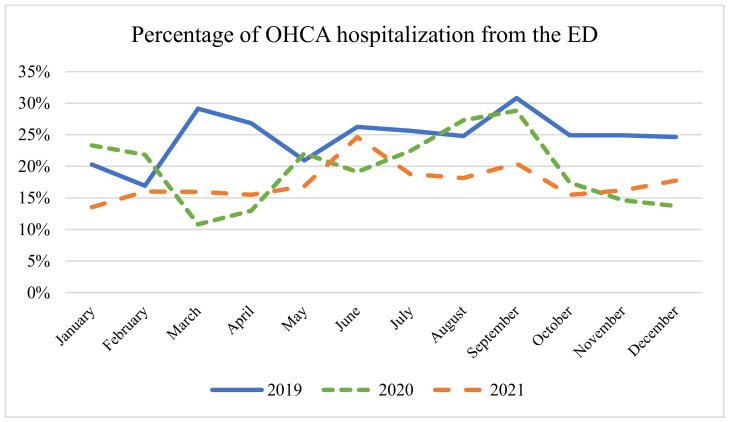
Percentage of OHCA hospitalizations from the ED. ANOVA test: monthly average 2019 (25%) vs. 2020 (19%) vs. 2021 (17%); *p* = 0.83.

Figure 4 illustrates the proportion of hospitalizations from the ED with a diagnosis of OHCA. There is a marked variability between the different years, with the average percentage standing at 25.7% in 2019, then decreasing to 19.5% in 2020 and 17.4% in 2021. These discrepancies are statistically significant, as indicated by the ANOVA test F(2, 33)= 8.97, *p* = 0.0007. Furthermore, the lowest percentage of hospitalizations in relation to the total number of admissions for OHCA was recorded during the first pandemic wave in March 2020, with a value of 10.8%.

## 4. Discussion

### 4.1. STEMI

Our analysis highlights a decrease in the incidences of STEMI diagnoses in 2020 compared to 2019, mirroring the findings of Rattka M. et al. [20]. Instead, in 2021 there is an increase in the number of STEMI diagnoses up to a return to pre-pandemic values (2019). Consistently with the analysis conducted by Pessoa-Amorim G. et al. [19], the lowest number of diagnoses was recorded in March 2020, with only 451 diagnoses compared to 754 in March 2019 and 722 in March 2021; this downturn coincided with the first pandemic wave. A second reduction in the number of STEMI diagnoses was observed during the second pandemic wave; as a matter of fact, in November 2020 there were 583 diagnoses, in contrast to 748 in 2019. Conversely, the average number of STEMI diagnoses remained unchanged during the third wave. No further analyses regarding the second or third waves are available in the existing literature.

The decline in the diagnostic frequency of STEMI diagnoses, concurrent with the waves of the pandemic, highlights how the stay-at-home policy has influenced patients’ decisions to seek medical treatment.

### 4.2. OHCA

Several factors can influence patient survival in OHCA [13]; among these, we have environmental conditions [13], the location of the event [34], layperson training [35], the proficiency and training of the advanced cardiovascular life support (ACLS) operators of the EMS system, and local policies [35,36,37]. Consequently, the observed increase in OHCA incidents may be attributable to various factors; however, in our investigation, the predominant influence of the COVID-19 pandemic on the healthcare system seems to be a relevant factor. 

Throughout the three reference years, we did not observe a significant alteration in the monthly incidences of OHCA diagnoses. However, we documented an increase in the number of OHCA diagnoses during the first wave. Two primary factors may have contributed to this phenomenon: firstly, OHCAs manifesting in patients experiencing respiratory distress associated with COVID-19; and secondly, patients with symptoms indicative of STEMI who decided not to seek treatment and stayed at home.

Figure 4 shows data that are rarely present in the existing literature. Notably, especially alongside the rise in the number of OHCA diagnoses during the first wave, there was a decreased percentage of individuals hospitalized after admission to the ED. This phenomenon may be attributed to two key factors. Firstly, given the spread of COVID-19, a substantial portion of these OHCAs may be attributable to hypoxic causes, which tend to present as conditions less amenable to reversal. Secondly, the transport and management times of these patients, as shown in the literature, appear to be longer, and therefore the likelihood of achieving ROSC could be sensitively reduced. Nevertheless, in 2021, we highlight the return to a percentage aligned with that of 2019. This observation constitutes significant data indicating a return to operational norms consistent with the pre-pandemic era. Indeed, the percentage of ROSC serves as a crucial indicator of the efficiency and functioning of the EMS system.

### 4.3. EMS

We observed a notable increase in the percentage of patients admitted to the ED and transported via 118 vehicles. Notably, during the pandemic phase, the Italian prime ministry repeatedly reiterated the importance of accessing the ED via 118 to ensure a correct clinical assessment and avoid overcrowding of the ED. This emphasis may explain the observed phenomenon. Our study analyzes the accesses in the EDs of the largest and most populous Italian region, taking into consideration a period of three years, which in turn allows us to analyze three pandemic waves and the inter-pandemic phases.

### 4.4. Relationship between OHCA and STEMI

During the COVID-19 pandemic, several studies have documented increased mortality rates in the general population [38,39,40]. As demonstrated in our analysis, two particularly pathophysiologically relevant diseases have markedly changed, which may be related to the increase in mortality. Understanding the inter-relationship between these two pathologies is crucial. Indeed, national health department directives advocating for a stay-at-home policy have led to a reduction in the number of ED accesses for relevant pathologies such as STEMI, coinciding with a surge in OHCA incidents. It is imperative to ascertain whether, in the post-pandemic era, the population continues to exhibit reluctance to access the ED. According to our analysis, this trend diminished. In 2021, there was an increase in STEMI accesses and a decrease in OHCA incidents, reverting to pre-pandemic levels.

In our opinion, as nations across the globe are actively engaged in formulating national pandemic plans [41], it is important to allocate attention to time-dependent pathologies. It is crucial to remember that the ED, even amidst a pandemic, must remain attractive for certain pathologies that, if not promptly treated, could lead to the death of the patient. Furthermore, a noteworthy aspect is the increase in the percentage of STEMI patients accessing the ED via EMS vehicles during the different phases of the pandemic. This phenomenon has been highlighted in various national healthcare systems [42,43] and, in our opinion, it is an aspect that needs further exploration. Notably, pandemic plans should also contemplate the possibility of increasing the number of rescue vehicles, given that, in many cases, family members or companions may be limited in accompanying their loved ones to the ED. This could potentially account for the observed increase in the percentage. In addition, this should also be achieved through appropriate simulation plans in order to support the analyses we have highlighted with the aim of explaining the roles and abilities of each emergency system operator [44,45]. Unfortunately, in our view, this element appears to be inadequately addressed in the formulation of national plans and should be implemented to a greater extent.

### 4.5. Strengths and Limitations

One of the strengths of our study lies in the time period analyzed, which includes one year of normal activity prior to the pandemic era (2019) and two years encompassing three different pandemic waves, the last of which occurred in early 2021; therefore, much of 2021 constitutes an additional inter-pandemic period. Another strength consists of the large sample size we were able to analyze, as the regional database includes access to 102 EDs in the Lombardy region.

This study has several limitations. Specifically, it is challenging to establish a definite correlation between the lack of access of STEMI patients to the ED and the increase in OHCA cases. Although there is a demonstrated correlation between the inadequate management of STEMIs within appropriate time frames and the development of OHCA, in our case, given that COVID-19 is a cause of hypoxia and can induce a generalized coagulopathy, the increase in OHCA cases could also be linked to an advanced development of such pathology. Additionally, during the various pandemic phases, due to the difficulties encountered in EDs, healthcare workers may have made a greater number of errors in documentation.

Authors should discuss the results and how they can be interpreted from the perspective of previous studies and the working hypotheses. The findings and their implications should be discussed in the broadest context possible. Future research directions may also be highlighted.

## 5. Conclusions

Under the three-year period, our analysis shows that during the pandemic peak, the overall number of STEMI diagnoses decreased, but the likelihood of receiving a STEMI diagnosis increased with total access; however, the number of STEMI diagnoses had reverted to the pre-pandemic trend within 2021. Concerning OHCA, we did not observe a significant alteration in the monthly incidence of OHCA diagnoses, except for an increase recorded during the first wave. Moreover, we observed a notable increase in the percentage of patients admitted to the ED and transported via 118 vehicles, which holds true both for the total number of accesses and, specifically, STEMI diagnoses.

As demonstrated in our analysis, population behavior changes during a pandemic, leading to a temporary change in the epidemiology of diseases. Additionally, governmental communications influence behaviors and alter the utilization of services by the population. Therefore, especially in situations that may restrict the free movement of the population, such as lockdowns, it is the government’s responsibility to convey messages correctly and, most importantly, to remind the population not to neglect any symptoms of time-dependent pathologies, such as STEMI. For this reason, our analysis shows how it is crucial to study the experiences from the past when drafting plans for the future.

## Data Availability

The data presented in this study are available on request from the corresponding author. The data are not publicly available in accordance with national data safety guidelines.
